# Residual Amino Acid Imbalance in Rats during Recovery from Acute Thioacetamide-Induced Hepatic Encephalopathy Indicates Incomplete Healing

**DOI:** 10.3390/ijms24043647

**Published:** 2023-02-11

**Authors:** Yevgeniya I. Shurubor, Alexander E. Rogozhin, Elena P. Isakova, Yulia I. Deryabina, Boris F. Krasnikov

**Affiliations:** 1 Centre for Strategic Planning of FMBA of Russia, Moscow 119121, Russia; 2 Valiev Institute of Physics and Technology of the Russian Academy of Sciences, Moscow 117218, Russia; 3 Bach Institute of Biochemistry, Research Center of Biotechnology of the Russian Academy of Sciences, Moscow 119071, Russia

**Keywords:** amino acids, HPLC, glutamine, glutamate, hepatic encephalopathy, enzymes

## Abstract

The delayed consequences of the influence of hepatic encephalopathy (HE) on the metabolism of animals have not been studied enough. We have previously shown that the development of acute HE under the influence of the thioacetamide (TAA) toxin is accompanied by pathological changes in the liver, an imbalance in CoA and acetyl CoA, as well as a number of metabolites of the TCA cycle. This paper discusses the change in the balance of amino acids (AAs) and related metabolites, as well as the activity of glutamine transaminase (GTK) and ω-amidase enzymes in the vital organs of animals 6 days after a single exposure to TAA. The balance of the main AAs in blood plasma, liver, kidney, and brain samples of control (n = 3) and TAA-induced groups (n = 13) of rats that received the toxin at doses of 200, 400, and 600 mg/kg was considered. Despite the apparent physiological recovery of the rats at the time of sampling, a residual imbalance in AA and associated enzymes persisted. The data obtained give an idea of the metabolic trends in the body of rats after their physiological recovery from TAA exposure and may be useful for prognostic purposes when choosing the necessary therapeutic agents.

## 1. Introduction

Hepatic encephalopathy (HE) is a neurodegenerative disease resulting from acute or chronic liver failure. The development of the disease occurs as a result of the intoxication of the liver and the entry of excess toxic substances into the blood. Clinical manifestations of HE also affect the brain, causing memory and psychomotor impairment, up to the development of coma or death [1].

To a certain extent, HE is considered to be a reversible disease. It was assumed that replacing the damaged liver could alleviate the neurological disease. However, this assumption was not confirmed in practice, with complications after liver transplantation persisting in half of the patients [2].

The pathogenesis, mechanisms, and consequences of the development of HE are still not entirely clear [3,4]. It is assumed that changes in brain metabolism are due to the development of hyperammonemia, which leads to an increase in the level of glutamine (Gln) in astrocytes that are responsible for the regulation of the blood–brain barrier (BBB) [3,4]. The dysfunction of the BBB, an increase in the level of Gln in astrocytes, and an imbalance in the content of a number of amino acids (AAs) can lead to cerebral edema and arterial hypertension [4]. The dysfunction of the immune and/or neurotransmitter systems under these conditions may act as a complicating factor [5,6,7].

As a result of the Gln metabolism and transamination of some AAs, up to 60% of ammonia can enter the body [8]. A number of authors emphasize the significant role of Gln in the development of HE. This is due to the fact that Gln in mitochondria is hydrolyzed to ammonia, which induces the mitochondrial pore (MPT), causing mitochondrial dysfunction and oxidative stress [9,10]. In addition to an increase in glycolytic activity and Gln synthesis in astrocytes, an impaired energy metabolism in the brain is often due to the inhibition of enzymes responsible for the synthesis of macroergs, ketoglutarate dehydrogenase (KGDH), and pyruvate dehydrogenase (PDH), and the dysfunction of the tricarboxylic acid cycle (TCA) [7,10,11].

Metabolic studies in the development of HE are often limited to assessments of the state of the liver or brain. At the same time, it is better to conduct such studies in a comprehensive manner, taking into account all vital organs: the liver, kidneys, brain, and circulating blood.

With the development of acute HE, a decrease in the level of branched-chain AAs (BCAAs) is usually noted, which support protein metabolism, apoptosis, hepatocyte regeneration, insulin resistance, and a number of other events [3,7,12,13,14]. BCAAs are actively absorbed by skeletal muscles and used for ammonia detoxification; therefore, the rate of their clearance in patients with liver cirrhosis is often associated with an increase in ammonia levels [13].

An increase in BCAA clearance shifts the AA balance towards an increase in the level of “toxic” aromatic amino acids (AAAs). It was noted that in patients with acute liver necrosis, there was a slight decrease (up to 80%) in the level of BCAAs against a marked increase in the level of AAAs and other AAs (Phe, Tyr, Met, Glu, and Asp) [15]. Soeters and colleagues believed that a decrease in BCAA levels is a consequence of hyperinsulinemia, and an increase in AAA levels indicates the presence of hyperglucagonemia [14]. At the same time, the ratio between pools of BCAAs (Leu, Val, and Iso) and AAAs (Phe, Tyr, and Trp), known as the Fisher coefficient [16], is sometimes used to indirectly assess liver function [3,7,12,14].

Some authors note that the development of HE in humans and animals under the influence of TAA has similar features [4]. The studied TAA rat models differ in doses (200–900 mg/kg), the duration of TAA use (hours/days/weeks/months), type of administration into the body (food/drinking water/intraperitoneal/intravenous injections), and frequency of exposure (single/multiple) [17,18,19,20]. Depending on the combination of the above factors in rats induced with TAA, up to five degrees of HE development can be distinguished, where the severity of the disease varies from a decrease in spontaneous activity, moderate ataxia, the absence of spontaneous movements, loss of reflexes, and coma [21,22].

The study of the recovery rate of rat liver after a single intraperitoneal injection of TAA at a dose of 500 mg/kg indicated its fairly rapid regeneration [23]. The results of the study of the morphology and biochemistry of rat liver indicated that the maximum cell death occurred in the first 24 h after the administration of TAA; however, the subsequent restoration of the hepatocyte population to a normal state and level occurred in 3 days after intoxication [23].

In general, low doses of TAA (200 mg/kg) did not cause significant liver damage in rats, while high doses (≥500 mg/kg) significantly reduced the survival of rats during the first 48 h. In this regard, for the optimal dose of TAA to obtain a HE model of rats, a dose close to 350 mg/kg was legitimized [24].

The development of HE affects not only the balance of AAs [9,10], but also the levels of energy metabolites, such as acetyl coenzyme A (acetyl CoA) [25,26,27], α-ketoglutarate (α-KG), α-ketoglutaramate (α-KGM), and others [28]. However, information on the balance of AAs and related metabolites after the completion of the acute stage of HE is limited. The aim of this study was to test the hypothesis that there is no statistically significant difference in the metabolic profiles of AAs in plasma, liver, kidneys, and brain tissues of TAA-induced rats followed by a 6-day postrehabilitation period and rats not exposed to TAA. In total, 16 amino acids were determined in blood plasma and tissue samples of the liver, kidneys, and brain, including aspartate (Asp), glutamate (Glu), glutamine (Gln), glycine (Gly), citrulline (Cit), arginine (Arg), taurine (Tau), alanine (Ala), tryptophan (Trp), methionine (Met), valine (Val), phenylalanine (Phe), isoleucine (Iso), leucine (Leu), ornithine (Orn), and lysine (Lys).

## 2. Results

### 2.1. Physiological State of Rats after TAA Intoxication

Twenty-four hours after receiving TAA at doses of 200 (n = 3), 400 (n = 6), and 600 (n = 4) mg/kg, the physiological state of the rats differed. At low concentrations of TAA (200 mg/kg), the animals had weak signs of depression, at the level of the first (mild) stage of HE development. Rats with higher doses of TAA (400–600 mg/kg) had symptoms similar to grades II/III (moderate/severe) of HE [22]. However, two rats that received a high dose of TAA (600 mg/kg) did not survive the first day after injection, which may be due to their individual response to the toxin. Two to three days after intoxication, the behavior of the animals did not visually differ from the behavior of the rats in the control group. Six days (remission) after the initiation of the acute phase of HE, the balance of sixteen AAs and the activity of two enzymes, GTK and ω-amidase, were assessed in the blood plasma and organs of rats.

### 2.2. Distribution of AA in Blood Plasma and Tissues of Rats

**Figure 1:**
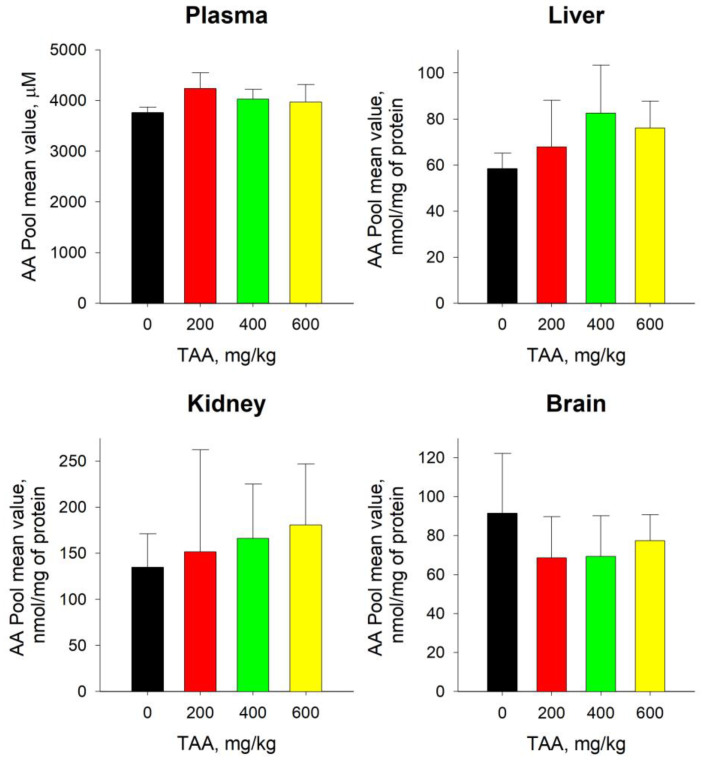
Sum of 16 amino acids in plasma, liver, kidney, and brain samples of control group of rats (n=3; black) and TAA-induced rats with IP doses of 200 (n = 3; red), 400 (n = 6; green), and 600 (n = 4; yellow) mg/kg. Data are expressed as group means ± standard deviation. Plasma AA levels are expressed in µM and tissue AA levels are expressed in nmol/mg of protein. No statistically significant differences were found.

The total level (sum) of AAs in the blood plasma of TAA-induced rats after their recovery remained somewhat higher than the level of the control group. The maximum excess (~12%) was noted in the blood plasma of rats that received minimal doses of TAA (200 mg/kg). In the blood plasma of rats that received higher doses of TAA (400 and 600 mg/kg), this excess was minimal, at ~7 and 4%, respectively (Figure 1).

**Figure 2:**
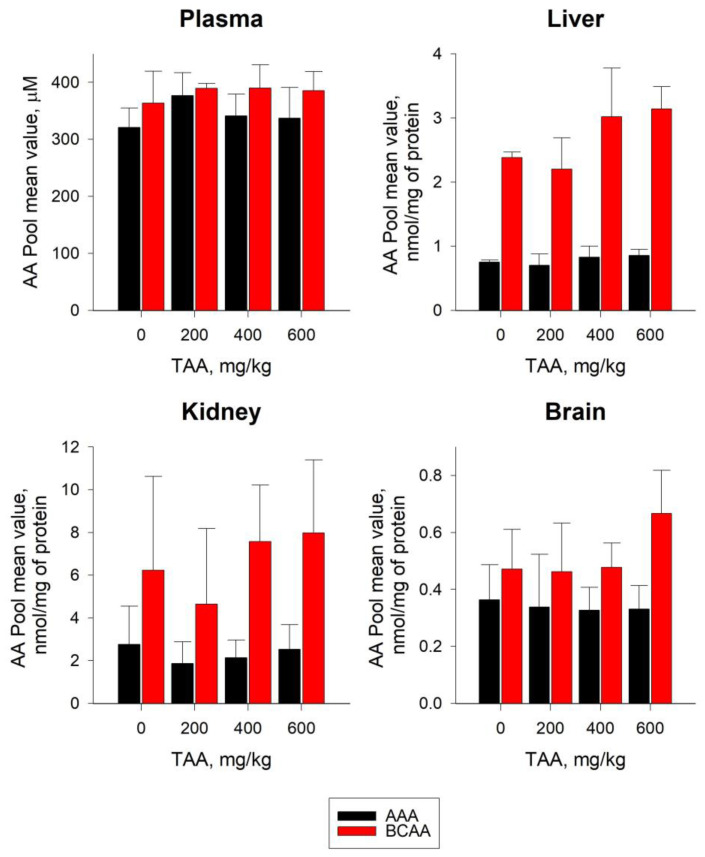
Sum of aromatic amino acid (black) and branched amino acid (red) levels in plasma, liver, kidney, and brain tissues of control group (n = 3) of rats and TAA-induced rats with IP doses of 200 (n = 3), 400 (n = 6), and 600 (n = 4) mg/kg. Data are expressed as group means ± standard deviation. Plasma AA levels are expressed in µM and tissue AA levels are expressed in nmol/mg of protein. Statistically significant differences between the control and TAA-induced groups for AAs of the same type were not revealed.

The levels of the majority (~75%) of individual AAs in the groups of TAA-induced rats exceeded the control values by ~3–30%. The concentrations of a minor quantity of AAs, namely, Asp, Ala, Glu, and Tau, were below the control values (~1–20%) (Table 1). Plasma levels of BCAAs and AAAs varied in a range of 360–390 and 320–380 µM, respectively. Moreover, in TAA-induced rats, the levels of BCAAs and AAAs were slightly higher (~6–7% and ~5–17%, respectively) than the control values (Figure 2).

**Figure 3:**
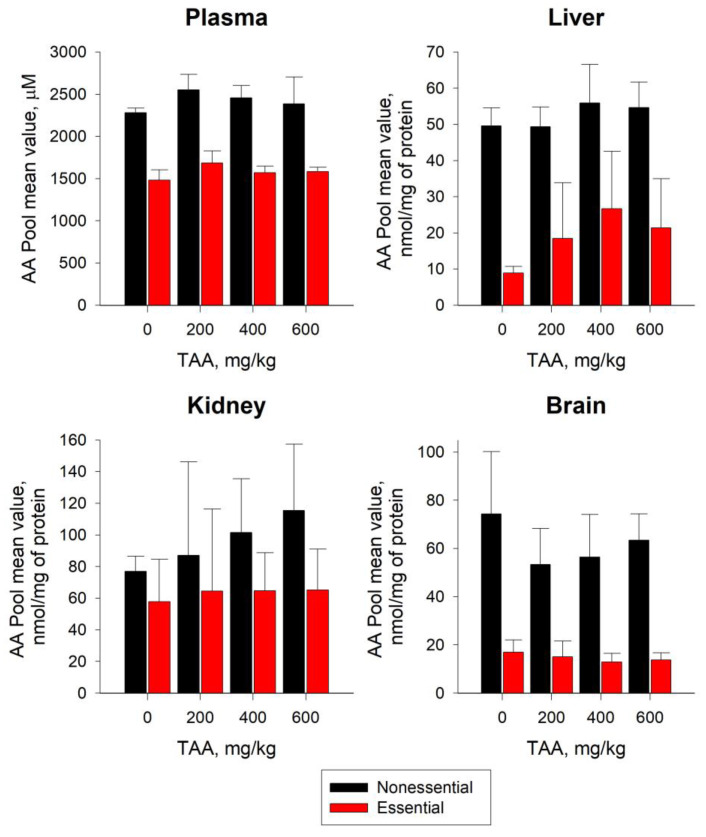
Sum of nonessential (black) and essential (red) AA levels in plasma, liver, kidney, and brain tissues of control group (n = 3) of rats and TAA-induced rats with IP doses of 200 (n = 3), 400 (n = 6), and 600 (n = 4) mg/kg, respectively, 6 days after their intoxication. Nonessential amino acids included Asp, Glu, Gln, Gly, Arg, and Ala. Essential amino acids included Trp, Met, Val, Phe, Iso, Leu, and Lys. Data are expressed as group means ± standard deviation. Plasma and tissue levels are expressed in µM and nmol/mg of protein, respectively. There were no statistically significant differences found between the control and TAA-induced groups for AAs of the same type.

Pools of essential AAs (Trp, Met, Val, Phe, Iso, and Leu) entering the body with nutrition and nonessential AAs (Asp, Glu, Gln, Gly, Arg, and Ala) synthesized in the body changed in blood plasma in ranges from 1500–1700 to 2300–2600 μM, respectively. In the plasma of TAA-induced rats, the pools of essential and nonessential AAs exceeded control values by ~6–14% and ~5–12%, respectively (Figure 3). In this regard, the ratio of nonessential/essential AAs in the plasma of TAA-induced rats decreased slightly relative to the control from 1.6 to 1.5 times (Figure 3).

The average values of the AA pools in the livers of TAA-induced rats also exceeded the control values by ~2–17% (Figure 1). However, in contrast to the dose-dependent distribution of AA pools in blood plasma, in the liver of rats, this distribution had the opposite direction (Figure 1). The levels of most of the individual AAs in the liver of rats exceeded the control values by ~5–45%, with the exception of Tau, which was significantly (≥4-fold) higher than the reference metabolite. The concentrations of five AAs, namely, Asp, Ala, Cit, Gly, and Trp, were below the control values by ~5–25% (Table 1). The Asp and Ala levels also showed a downward trend in plasma.

In the liver of TAA-induced rats, in contrast to blood plasma, there was a significant (up to ~3–3.5 times) excess of BCAA levels over AAA levels (Figure 2). In the liver of rats given a low dose of TAA (200 mg/kg), AAA and BCAA values were comparable to control values. In the liver of rats with higher (400–600 mg/kg) doses of TAA, there was an increase in the levels of both AA pools. Moreover, the increase in BCAA levels was ~25–35% relative to the control and was almost twice as high as the increase in AAA levels (~10–15%). The sum of essential and nonessential AAs in the rat livers ranged from 9–27 to ~50–55 nmol/mg protein, respectively. The increase in the sum of essential AAs (~ up to 2–3 times) in the livers of TAA-induced rats relative to the control values was ~15–20-fold as high as the increase in nonessential AAs (~up to 13%) (Figure 3). Despite the increase in AA levels in the groups of TAA-induced rats, the ratio of nonessential/essential AAs decreased from ~6 times in the control to ~2.5 times in the TAA groups, which exceeded the similar ratio in the blood plasma of rats (1.5–1.6 times) (Figure 3).

The absolute content of the AA pools in the tissues of the kidneys of rats was ~2–3 times higher than in the liver (Figure 1) and increased with an increase in the dose of TAA. In the groups of rats that underwent TAA intoxication at doses of 200, 400, and 600 mg/kg, the increases relative to the control were ~11, 19, and 27%, respectively (Figure 1). The levels of individual AA pools in the kidneys of TAA-induced rats exceeded the control values by ~4–77% (Table 1). The maximum increase (~ up to 5-fold) was noted for Val, and the maximum decrease for Phe and Leu (~ up to 2–3-fold).

The levels of BCAAs and AAAs in the tissues of the kidneys of rats with a low dose of TAA (200 mg/kg) were lower than the control by (~25% and ~30%), respectively. At higher doses of TAA (400 and 600 mg/kg), the BCAA concentrations increased by ~20–30% and the AAAs decreased by ~10–20%, respectively (Figure 2). Essential and nonessential AA pools in the kidneys ranged from ~60–65 to ~75–115 nmol/mg protein, respectively. In the kidneys of TAA-induced rats, an increase in the pools of essential (~12–13%) and nonessential (~15–50%) AAs was also observed (Figure 3). The ratio of nonessential/essential AAs varied from 1.3 times in the control samples to 1.8 times in the TAA-induced groups of rats.

In the brain tissues of TAA-induced rats, the AA pool was reduced by ~20–35% compared to the control. Moreover, the level of the AA pools in rats that previously received the minimum and average doses of TAA (200 and 400 mg/kg) was lower than the control values by ~35–32%, and in rats that received the maximum doses of TAA (600 mg/kg), such a decrease was at the level of ~18% (Figure 1). Additionally, unlike other samples, in the brain tissues of TAA-induced rats, there was a decrease in the levels of most individual AAs by ~2–35%. Only four AAs, namely, Arg, Val, Leu, and Orn, were ~4–27% higher than the control values.

The pattern of the distribution of AAAs and BCAAs in rat brain tissues also differed from that in blood plasma, liver, and kidneys. AAA values decreased by ~7–10% relative to the control with an increasing TAA dose, while BCAA values, on the contrary, increased up to ~40%, especially in the group with the highest TAA dose (Figure 2). In the brain tissues of TAA-induced rats, the level of essential and nonessential AAs decreased, relative to the control, by ~10–30% (Figure 3). In rat brain tissues, an average sum of essential AAs varied from 13 to 17 nmol/mg of protein; moreover, in the group of TAA-induced rats, a ~10–25% decrease relative to the control was observed (Figure 3). The level of nonessential AAs was significantly higher, and ranged from ~55 to 75 nmol/mg of protein, with a gradual decrease (by ~15–30%) from the control group to the high-dose TAA group. In rat brain tissues of TAA-induced rats, the nonessential/essential AA ratio varied within ~3.5–4.5, which was almost two times higher than similar ratios in blood plasma, liver, and kidney tissues.

Thus, the minimum difference between the essential and nonessential AA pools was noted in the blood plasma of rats (~1.5 times), and the maximum difference was observed in the tissues of the liver (up to ~2.5–5.5 times), brain (up to ~3.5–4.5 times), and kidneys (up to ~1.3–1.8 times) (Figure 3).

### 2.3. Glutamine and Glutamate in Blood Plasma and Tissues of Rats

Glu plays a special role in the regulation of the peripheral and central nervous systems. Under HE conditions, changes in glutamatergic neurotransmission, responsible for the deterioration of the patient’s mental abilities, were noted [29].

In the blood plasma of rats, after the completion of the acute HE phase, the Gln level was slightly higher (~15%) for the control values (Table 1 and Figure 3), and the Glu level was lower (~20%). In this regard, the ratio of Gln/Glu in the blood plasma of the control group of rats was equal to five, and in the group of TAA-induced rats it was ~10–70% higher. In the liver tissues of TAA-induced rats, Glu and Gln levels were ~5% and ~35% higher than control values, respectively (Table 1 and Figure 3). The ratio of Gln/Glu in the liver of rats of the control group was two-fold, and in the liver of experimental rats it increased by ~15–70%. In the kidney tissues, the levels of Glu and Gln in the group of TAA-induced rats exceeded the control values by ~30–50% (Table 1 and Figure 3). The Gln/Glu ratio was minimal here, and amounted to ~0.2 in the control and ~0.3 in the group of TAA-induced rats. This circumstance, apparently, was due to the increased growth of Gln to almost twice the levels of the growth of Glu (Table 1 and Figure 3). In the brain tissues of TAA-induced rats, as well as in the kidneys, the levels of the Gln/Glu ratio were low and varied from 0.4 in the control to 0.5 in the group of TAA-induced rats. In contrast to the tissues of the livers and kidneys, the levels of Glu and Gln in the groups of TAA-induced rats were reduced relative to the control by ~20–30%, where the decrease in Glu was more pronounced.

### 2.4. GTK and ω-Amidase Activity in Blood Plasma and Tissues of Rats

Due to the fact that GTK was not present in the blood plasma of rats (in contrast to tissues), only the activity of ω-amidase was determined in these samples.

**Figure 4:**
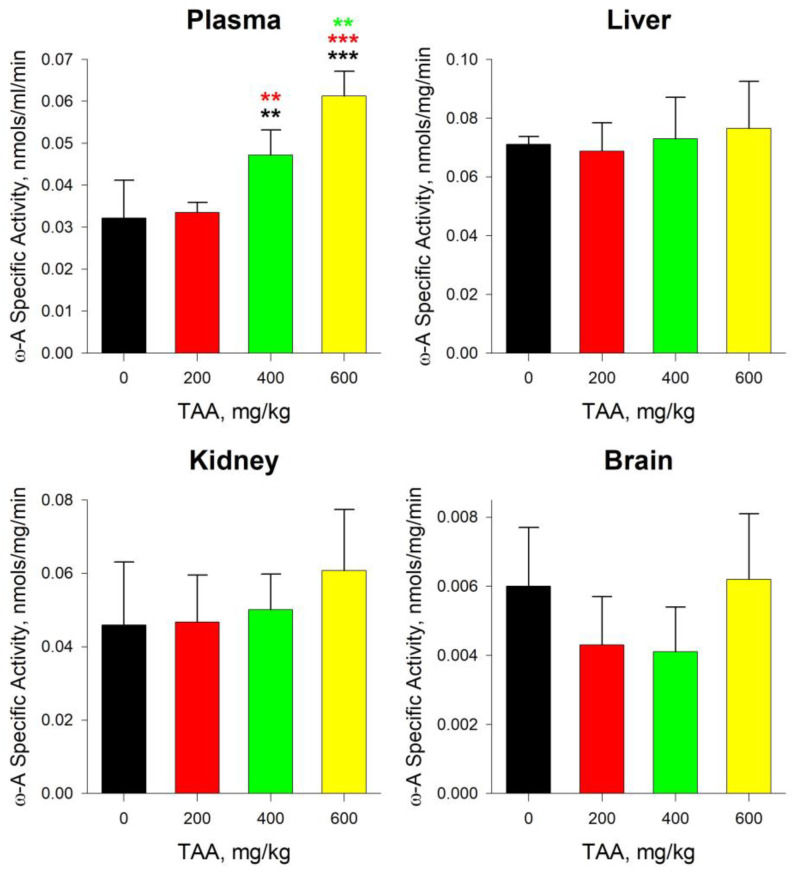
The levels of specific activity of ω-amidase in the tissues of the liver, kidneys, brain, and blood plasma in the control (n = 3; black) and TAA-induced groups of rats at intraperitoneal doses of TAA 200 (n = 3; red), 400 (n = 6; green), and 600 (n = 4; yellow) mg/kg. Data are presented as mean ± standard deviation and expressed in nmole/mg/min. Statistically significant differences were found for the TAA 400 and 600 groups compared with the control (two and three stars marked in black, *p* ≤ 0.05 and ≤0.01, respectively), the TAA 400 and 600 groups compared with the TAA 200 group (two and three stars marked in red, *p* ≤ 0.05 and ≤0.01, respectively), and TAA 600 compared to TAA 400 (two stars marked in green, *p* ≤ 0.05).

The absolute values of ω-amidase activity in the blood plasma of TAA-induced rats gradually increased from 0.032 nmole/mg/min in the control to 0.061 nmole/mg/min in the group with the maximum dose of TAA (Figure 4). The activity of ω-amidase in the control samples of rat livers was almost twice that in blood plasma. However, the dependence of the activity of this enzyme on the dose of the received TAA changed insignificantly from 0.071 nmole/mg/min in the control to 0.077 nmole/mg/min (~10%) in the experiment group.

**Figure 5:**
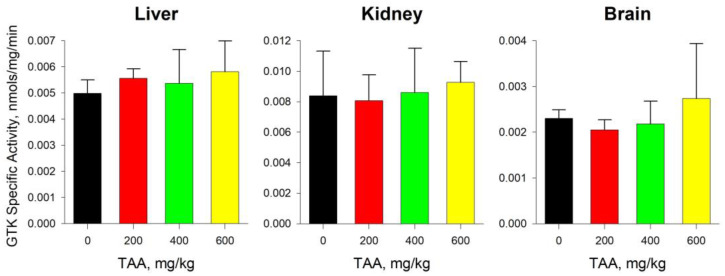
The levels of GTK-specific activity in the tissues of liver, kidneys, and brain of control group (n = 3; black) of rats and TAA-induced rats with IP doses of 200 (n = 3; red), 400 (n = 6; green), and 600 (n = 4; yellow) mg/kg. Data are presented as mean ± standard deviation and expressed in nmole/mg/min. No statistically significant differences were found.

The activity of GTK in the liver of the control group of rats was almost 15 times lower than the activity of ω-amidase, and amounted to approximately 0.005 nmole/mg/min. With increasing doses of TAA administration, the GTK activity increased slightly to 0.006 nmole/mg/min (~17%) in the high-dose TAA group of rats (Figure 5).

The absolute values of the activity of ω-amidase in the tissues of the kidneys were intermediate between the activity of the enzyme in the blood plasma and in the tissues of the liver. In the kidneys of the control group of rats, ω-amidase activity was 0.046 nmole/mg/min and increased to 0.061 nmole/mg/min (≥30%) with the increasing dose of TAA (Figure 4). The difference between the activity of GTK and ω-amidase in the tissues of the kidneys was not as significant as in the liver, where the activity of GTK was approximately six times lower than the activity of ω-amidase. It was assumed that this may have been due to the higher initial activity of GTK in the kidneys of rats, exceeding the activity of GTK in the liver by almost ~70%. In general, GTK activity in the control and experimental groups of rats changed insignificantly, from 0.008 nmole/mg/min to 0.009 nmole/mg/min (~10%).

The activity of both enzymes in rat brain tissues was the lowest. The activity of ω-amidase in the brain tissues of the control groups of rats was lower than in the blood plasma, liver, and kidney tissues by ~5, ~12, and ~8 times, respectively. GTK activity changed to a lesser extent and was ~2 and ~4 times lower than in the liver and kidneys. The activity of both enzymes in the brain tissues changed disproportionately with the increasing doses of TAA. Thus, in the groups of rats with low and medium (200–400 mg/kg) doses of TAA, the activity of both enzymes was lower than the control values, and in the group of rats with a high dose (600 mg/kg) of TAA, it was higher (Figure 5). In general, the distribution of enzymatic activity in groups of rats with different doses of TAA corresponded to the distribution of the total pools of AAs, including Glu and Gln (Figure 1, Figure 3, Figure 4 and Figure 5).

### 2.5. Statistical Analysis

The relationship between (a) the concentration values of sixteen AAs in different types of blood plasma, liver tissue, kidneys, and brain samples and (b) groups of rats treated with TAA doses of 0, 200, 400, and 600 mg/kg was investigated.

In total, 1953 tests for the normality of the two-dimensional distribution of AA concentrations in various organs were performed using the mshapiro_test function, 1953 correlation estimates using the cor_test function, 1953 tests for the homogeneity of covariance matrices using the box_m function, and 256 tests for the equality of variances using the bartlett.test function. Estimated correlations (cor_test function) with a significance level of ≤5% between AA variations in samples of the same type are given as an example in Table S1 of the Supplemental Materials S1. The levels of correlations between the metabolites of different types of samples were not included in the table.

From the generalized calculation data given in Table S1 of the Supplemental Materials, it follows that the largest number of paired AAs with a significant correlation was found in brain tissues (*p* value ≤ 0.05, mean value 0.011, and n = 34). Slightly lower amounts of paired AAs were found in the kidneys (*p*-value ≤ 0.044, mean 0.013, and n = 32) and livers (*p*-value ≤ 0.05, mean 0.015, and n = 26), as well as in blood plasma (*p*-value ≤ 0.05, mean 0.021, and n = 10). Based on the calculated data, one could make an assumption about the degree of resistance of each of the studied organs to the effects of the toxin. Judging by the largest number of detected paired AAs with high correlation, the brain was the most resistant to the effects of TAA. Blood plasma had the least stability, where the number of paired AAs with a correlation of ≤0.05 was three times less than in brain tissues, and the average level of correlation was twice as high.

After the above check, a two-dimensional analysis of variance of AA concentrations from one type of tissue for the pairs of dependent variables (MANOVA) was performed. For ten AAs, the difference in concentration was significant at the level of 5% (Supplemental Materials, Table S2). Finally, for these AAs, Tukey’s pairwise test was used to identify a statistically significant difference in AA concentrations between all experimental groups of rats (Supplemental Materials, Figures S1–S10).

## 3. Discussion

With the development of acute HE, toxins from the circulating blood penetrate the liver, kidneys, and sometimes the brain, causing a shift in the metabolic balance. The amount of ammonia in the body is influenced by the process of the catabolism of amino acids obtained with dietary protein. Arg, Asn, Gln, His, Lys, and Met have the highest potential for this [30].

The main site of the metabolism of most AAs is the liver, which, under conditions of HE, supplies elevated concentrations of AA to the blood [15]. The development of HE and the imbalance in the AA content are reversible and can be corrected with the use of AA-containing supplements or restorative procedures, such as infusional therapy, plasma exchange, or dialysis [15,31].

### 3.1. AA Pools in Blood Plasma and Tissues of Rats

In general, the trends in the distribution of the AA pools in the liver and kidneys of rats with increasing doses of TAA were similar, whereas in the blood plasma and brain of rats, they were rather multidirectional (Figure 1).

In the blood plasma of rats, the levels of BCAAs and AAAs differed slightly from one another, although in the groups of TAA-induced rats, a slight increase relative to the control values was noted. A certain increase in the groups of TAA-induced rats was also noted for essential and nonessential AAs. At the same time, the upward trend in the accumulation of essential AAs in groups of rats with higher doses of TAA was more pronounced than nonessential AAs. A similar trend was noted earlier in the development of acute HE, mainly affecting such metabolites as Phe, Tyr, Met, Glu, and Asp [15].

Information about the redistribution of the balance of individual AAs as a result of the development of acute HE is of practical interest. Despite the lack of publications on this topic, it is worth considering the work of Fontana et al. [12]. This publication notes that an increase in the blood concentrations of Gly, Val, Met, Tau, Glu, Lys, Orn, Phe and Cit in the blood of TAA-induced animals is accompanied by a decrease in the level of Asp.. Other authors noted an explosive (up to 27 times) increase in Met concentrations in patients with acute fulminant liver failure [32].

Our data indicate the maintenance of elevated levels of Phe, Met, Gly, Val, Lys, Orn, Gln, Arg, Trp, Iso, Leu, and Cit in the blood plasma of rats undergoing HE, as well as reduced levels of Glu, Asp, Tau, and Al. Attention was drawn to the presence of similar trends in the distribution of the total pools of AAs (Figure 1) and acetyl CoA, previously determined in the same blood plasma samples [27]. Acetyl CoA is one of the key metabolite regulators of energy metabolism in cells, and the similarity in these trends could indicate a close relationship between the balance of AA levels and the TCA cycle.

In the blood plasma of TAA-induced rats, compared with the control group, there was a slight increase in the levels of BCAAs and AAAs (~6–7% and ~5–17%, respectively). In the groups of TAA-induced rats, a slight increase in essential (~6–14%) and nonessential AAs was also noted (~5–12%).

The predominant oxidation of AAAs occurs in the liver, while the oxidation of BCAAs occurs in muscles [33,34]. Serious liver pathologies are often accompanied by some decrease in BCAA levels and an accumulation of AAAs. Moreover, liver dysfunction affects the rate of BCAA oxidation much less, so their level is less susceptible to fluctuations [13,14,15,34,35].

In the livers of rats that recovered after receiving high doses of TAA, the level of AA pools remained elevated. In groups of rats with low doses of TAA (200 mg/kg), the content of the AA pools was close to control values, which, apparently, indicated their faster recovery. The obtained data coincided with the conclusion about the absence of significant damage and a faster recovery of the livers of rats at low doses of the obtained TAA [24]. As follows from Figure 2, the levels of BCAA accumulation in the liver were several times higher than the levels of AAA accumulation, which here may have also been a consequence of the presence of certain stabilization processes.

In the liver tissues and blood plasma of TAA-induced rats, elevated levels of essential and nonessential AA were noted. However, if the increase in nonessential AAs in the liver of TAA-induced rats, relative to the control, was ~10–13%, then the increase in essential AAs was ~2–3-fold. Such an accumulation level of nonessential AAs could indicate a slight lag in the synthesis of AAs from their supply from outside. As noted earlier, the supplementation of essential AAs in animal diets could improve liver health in post-HE rats, restore antioxidant marker levels, and reduce the effects of inflammatory processes [36].

TAA toxicity reduces the ability of the kidneys to filter blood effectively, and high doses of the toxin can cause organ failure or death. In the kidneys, compared with the liver, the content of the AA pools was almost twice as high (Figure 1). The increase in the AA pools in the kidneys of TAA-induced rats, relative to the control values, was almost a third higher than in the liver. Moreover, the greatest excess of AA pools in the kidneys relative to the liver was noted in the group of rats with the minimum dose of TAA (200 mg/kg). This circumstance could indicate a higher vulnerability of the kidneys (even at low doses of the toxin) compared to the liver, or their slower recovery after undergoing HE.

In the tissues of the kidneys of postrehabilitation rats, there was a decent decrease of up to ~25–30% in the levels of AAAs and BCAAs in groups with low doses of TAA, which was accompanied by a noticeable increase in BCAAs of up to ~30% in groups of rats with medium and high doses of TAA, and about the same decrease (up to ~25%) in AAAs.

The control level of AA pools in brain tissues was higher than in liver tissues, but lower than in the kidneys (Figure 1). In contrast to the tissues of the liver and kidneys, the accumulation levels of AAs in the brain tissues of TAA-induced rats were downward rather than upward (Figure 1). That is, if there was a slight increase in the pools of AAs in the tissues of the liver, kidneys, and blood plasma of rats with increasing doses of TAA, then the brain tissues would show a slight decrease. Perhaps this was due to the selective “capture and retention” of AAs by tissue cells and their relatively weak release into the circulating blood.

It was noted that when entering the brain, BCAAs and AAAs compete for the same transport protein, while BCAAs are involved in protein synthesis, neurotransmitters, and energy production [37]. The recovery period of rats after exposure to TAA was accompanied by a slight increase (up to ~40%) in the content of BCAAs alongside a decrease in AAAs (~7–10%). The pools of essential and nonessential AAs in the brain tissues of TAA-induced rats decreased by ~10–30% relative to the control, while the prevalence of the pools of nonessential AAs over essential AAs remained high and was ~4.5-fold.

### 3.2. Glu and Gln in Blood Plasma and Tissues of Rats

In brain tissue, unlike the liver and kidneys, there is no efficient urea cycle. The Gln–Glu cycle, which can undergo significant changes, is responsible for the process of ammonia detoxification during the development of HE. Ammonia and astrocytes play a fundamental role in the development of HE, affecting the intercellular transport (uptake and release), synthesis, and functioning of the Gln–Glu cycle in the brain [38]. Gln and Glu are directly involved in the stabilization of cellular homeostasis and ammonia detoxification [8]. At the same time, the Gln molecule is a neutral AA, a nontoxic carrier of ammonia in the CNS, the synthesis of which is aimed at protecting astrocytes in the event of an increase in the concentration of ammonia in the blood. Glu is an excitatory neurotransmitter in the CNS and modulates brain processes such as encoding information, forming and searching for memories, spatial recognition, and the maintenance of consciousness [39].

It was noted that in the brain tissues of rats during the development of TAA-induced HE, a decrease in the level of Glu was observed [39,40]. In the brain tissues of patients with hepatic comas, a decrease in the Glu concentration was also noted, which may have been due to its reverse astrocytic absorption associated with a decrease in the capacity of the astrocytic transporter [41]. A significant surge in serum transaminase activity was also noted in animals after TAA induction, followed by a complete stabilization within a day [40].

Generally, in the blood plasma, liver, and kidney tissues of TAA-induced rats after their recovery, the levels of Gln exceeded the control values, while being reduced in the brain tissues. In blood plasma, for example, the increase in Gln in the groups of TAA-induced rats was accompanied by a proportional decrease in the level of Glu (Figure 3). Moreover, the highest level of Gln was noted in the group of rats with a low dose of TAA and gradually decreased with an increase in the dose of TAA. The distribution of Glu in the same groups of rats had an opposite trend: in the groups with a low dose of TAA, the level of Glu was minimal and gradually increased with an increase in the dose of TAA. As a result, the ratio of Gln to Glu in all TAA-induced groups of rats, and especially in the group with low doses of TAA, significantly exceeded the control values (Figure 4).

The increased concentrations of Glu and Gln in the liver and kidneys of TAA-induced postrehabilitation rats may have been due to their participation in the processes of intermediate metabolism and reabsorption. Here, the Gln/Glu ratio was also higher in the liver and kidney tissues of TAA-induced rats. Moreover, higher ratios were found in groups of rats with low and medium doses of TAA.

In contrast to the tissues of the liver and kidneys, the concentrations of Gln and Glu in the brain tissues of TAA-induced rats were lower than the control values. However, the decrease in the level of Glu here was more pronounced than that of Gln. It should be noted that the trends in the dose-dependent distribution of Glu in blood plasma, liver, kidney, and brain tissues of rats generally coincided (Figure 3). In contrast to Glu, the distribution of Gln in brain tissues differed from its distribution in other samples; it was rather reversed, which may indicate a slight shift in the balance towards the increased utilization of Gln. This assumption could be supported by data on the distribution of blood plasma and tissues of α-KG and α-KGM [42], the direct participants in the Gln–Glu cycle, in the blood plasma and tissues of rats. According to the data obtained, an increase in the dose of TAA was accompanied by an almost two-fold increase in the level of α-KG in brain tissues with a similar decrease in the level of α-KGM.

Usually, the Gln catabolism begins with the conversion of Gln to Glu and its conversion to α-KG. However, there is another pathway in cells, when ammonia is formed from the amide position of Gln. This is the glutaminase II pathway, consisting of GTK, which catalyzes the transamination of Gln into the neurotoxin α-KGM, and ω-amidase, which catalyzes the hydrolysis of α-KGM to α-KG and ammonia [43]. The GTK-catalyzed formation of α-KGM from Gln is reversible, but its further binding to ω-amidase makes the transamination reaction irreversible [43]. Under physiological conditions, α-KGM is rapidly removed as a result of the cyclization of the molecule or its transformation into α-KG, while GTK plays an important role in preventing the accumulation of α-keto acids resulting from nonspecific aminotransferase reactions [44]. Most often, α-KGM is represented by the cyclic form, while ω-amidase has a greater affinity for the open, linear form of α-KGM, which is more easily formed at a pH close to 8.5. The ability of ω-amidase to convert toxic substrates, such as α-KGM, into bioenergetically important metabolites, such as α-KG, allows it to be classified as a repair enzyme [45].

In the brain tissues of TAA-induced rats, there was a decrease in the levels of Gln, GTK, and ω-amidase in the groups of rats with lower doses of TAA (200 and 400 mg/kg) and an increase in the group with a high dose of TAA (600 mg/kg). Trends in the distribution of Gln, GTK, ω-amidase, and α-KGM levels were rather inversely proportional (Figure 3, Figure 4 and Figure 5).

It should be noted that increasing the dose of TAA led to a decrease in the levels of α-KGM in all rat samples [42]. At the same time, a decrease in the levels of α-KG was observed in the blood plasma and in the tissues of the kidneys of TAA-induced rats, while its levels increased in the tissues of the liver and brain. In the blood plasma and in the tissues of the kidneys, the distribution of α-KG and α-KGM with an increase in the dose of TAA was unidirectional, and in the tissues of the liver and brain of rats, it was multidirectional. It is possible that during the recovery period of rats after acute HE, this may be due to the use of various alternative pathways for the conversion of both Glu and Gln.

In general, the data obtained indicate that the nature of the distribution of Glu in the samples of TAA-induced rats during the period of their rehabilitation coincided with the distribution of ω-amidase and GTK in them (Figure 3, Figure 4 and Figure 5). The direction of a number of enzymatic reactions during the synthesis and catabolism of Glu and Gln could be determined with the pH balance in cells. For example, as one of the regulators of the acid–base balance, the dysfunction of the liver during the development of HE can cause complex acid–base anomalies and a redirection of Gln and Glu fluxes in the tissues of the kidneys, liver, and brain of rats [46]. At the same time, a decrease in the pH in the kidneys stimulates the Gln and Glu metabolism, internal Gln transport, and external Glu transport, while an increase in the pH leads to the reverse processes. At the same time, an increased absorption of Glu can be observed in brain astrocytes [47].

## 4. Materials and Methods

### 4.1. Chemicals

All AAs of the highest purity standards, including potassium carbonate, sodium acetate (trihydrate), potassium phosphate, 2-mercaptoethanol, o-phthalaldehyde, and tetrahydrofuran, were obtained from Sigma-Aldrich (St Louis, MO, USA). Perchloric acid (PCA, 69–72% W/V) was obtained from J.T. Baker (Phillipsburg, NJ, USA). Brij-35 was obtained from Santa Cruz Biotechnology (Dallas, TX, USA). Methanol was obtained from Bruker, (Billerica, MA, USA). Ultrapure water was obtained using a Milli-Q Gradient A10 system (Millipore, Bedford, MA, USA). All chemicals were HPLC-grade purity and used without further purification. A nylon membrane filter with a diameter of 47 mm and pore size of 0.2 µm was obtained from Pall Life Science (Port Washington, NY, USA) and used for mobile-phase filtering and degassing.

### 4.2. Rat Model of Liver Failure

Eighteen 4-month-old female outbred Wistar rats weighing 130–140 g were used in the experiment. For 2 weeks before the start of the experiment, they were quarantined with a 12 h light cycle, receiving normal food and drinking water.

TAA for injection was prepared in saline and sterilized through a 0.2 µm Millipore filter. The control group of rats (n = 3) received a single intraperitoneal injection of sterile saline, and the remaining rats received doses of TAA in the amounts of 200 mg/kg (n = 3), 400 mg/kg (n = 6), and 600 mg/kg (n = 6; two animals did not survive the treatment). Blood plasma and organ samples from rats were frozen in liquid nitrogen 6 days after they received doses of TAA.

The maintenance of the animals, the procedures performed, and the experimental protocols complied with the Animal Protocols No. 22/1 approved by the supervisory board of the Bach Institute of Biochemistry and by the bioethics committee of the Biotechnology Research Center of the Russian Academy of Sciences, Moscow.

### 4.3. AA Measurements and Sample Preparation

The measurement of AAs in biological samples was carried out with HPLC [48], with minor modifications. Briefly, a Waters 474 multiscan fluorescent detector, a Waters 717-plus autosampler with a cooled platform (+4 °C), and a Perkin-Elmer 250 binary system were used. For the separation of AAs, a Supelco C18 analytical column, 4.6 mm × 15 cm, 3 µm, with a YMC C18 guard column, 4.6 mm × 5 cm, 15 µm, was used. Data collection, analysis, and archiving were controlled with ESA501 software installed on a Dell computer. Fluorescence was monitored at excitation and emission wavelengths of 340 and 455 nm, respectively. Precolumn derivatization was performed using a Waters 717-plus autosampler. A sample delivery was performed using a gradient profile with two mobile phases. For mobile-phase A, 27.3 g sodium acetate (trihydrate) dissolved in 2.0 L H_2_O, 96 µL of 6 N HCl, 180 mL methanol, and 10 mL tetrahydrofuran (pH 7.2) was used. Mobilephase B was 100% pure methanol. Both mobile phases were filtered through 0.2 µm nylon membrane filters before use [48]. The final concentration of metabolites in the standard mixture was 40 μM, except for Trp, Met, Val, and Phe, which had a final concentration of 10 μM.

### 4.4. Tissue Samples Were Prepared as Follows

To a ~50 mg frozen tissue sample, 500 µL of 1.5 M ice-cold perchloric acid (PCA) was added. Brain tissues were homogenized through sonication for 6 sec with an amplitude of 15%, and the liver/kidney tissues were homogenized through sonication twice for 6 sec with an amplitude of 20%. During the sonication, as well as for 3–5 min thereafter, the samples were kept on ice with periodic vortexing. Then, 500 µL of double-distilled water was added to the obtained homogenate, mixed well, and neutralized with 250 µL of 2 M K_2_CO_3_ (added slowly with precaution). A portion of the obtained homogenate (20–25 µL) was saved for a further protein analysis if needed. The obtained homogenate was centrifuged for 10 min at 14,000 g (+4 °C). Then, the supernatant was transferred to a new Eppendorf tube and centrifuged again, as described above. After the second centrifugation, 25 µL of 1.2% benzoic acid and 350 µL of double-distilled water were added to a combined 25 µL of a clear supernatant and 25 µL of a mixed AA standard solution. The rest of the undiluted sample was saved and stored at −80℃.

### 4.5. Plasma Samples Were Prepared as Follows

To 50 µL of plasma, 50 µL of 1.5 M ice-cold PCA, 375 µL of double-distilled water, and 25 µL of 2 M K_2_CO_3_ (mixed well after each step) were added. The obtained solution was centrifuged for 10 min at 14,000 g (4 °C). The supernatant was transferred to a new Eppendorf tube and centrifuged again, as described above. After the second centrifugation, 25 µL of a clear supernatant and 25 µL of a mixed AA standard solution were combined, followed by the addition of 25 µL of 1.2% benzoic acid and 350 µL of double-distilled water. The rest of the undiluted sample was saved and stored at −80 °C.

### 4.6. OPA Solution for Sample Derivatization

The OPA *o*-phthaldialdehyde solution for the sample derivatization was freshly prepared through proportional OPA dilution (25 mg) in pure methanol (0.63 mL) with the addition of 40 mM of a Na–borate solution (5.6 mL), β-mercaptoethanol (24 µL), and 30% Brij (0.64 mL) [48], with brief mixing after each step. The obtained OPA solution was stored on ice and used within 36 h of preparation. A single sample analysis required 50 min of HPLC use. We then routinely analyzed 18–20 biological samples and 2–3 mixed AA standard solutions a day, inserted at the beginning, in the middle, and at the end of each run. The protein level in tissues was measured using the Biuret method; the metabolite concentration in the samples was calculated based on the authentic external standard concentration.

### 4.7. Determination of ω-Amidase and GTK Activity

The measurement of enzyme activity in blood plasma and animal tissue samples was carried out with spectrophotometric methods developed for 96-well plates, as described below.

#### 4.7.1. ω-Amidase Endpoint Assay

The reaction mixture (RM) contained 5 mM α-KGM, 5 mM DTT, 100 mM Tris-HCl buffer (pH 8.5), and an enzyme source. The final RM volume was adjusted with H_2_O to 50 µL. Note that when the assays were conducted with purified ω-amidase, the blank contained a complete RM lacking the enzyme. For assays of crude tissue/cell homogenates, the blank contained a complete RM plus a homogenate and 200 mM of glycylglycine. After a 5–30 min incubation at 37 °C, the reaction was terminated by the addition of 20 µL of 5 mM 2,4-dinitrophenylhydrazine in 2 M HCl. After a further incubation for 5 min at 37 °C, 130 µL of 1 M NaOH was added and the absorbance was read at 430 nm within 5 min. The ε_430nm_ of α-ketoglutarate*2,4-dinitrophenylhydrazone under these conditions was 16,000 M^−1^·cm^−1^.

#### 4.7.2. GTK Endpoint Assay

The RM for the assay contained 50 mM ammediol (Amm), 2.5 mM α-keto-γ-methiobutirate (KMB), 10 mM L-phenylalanine (Phe), and a 5 µL sample. The total RM volume was adjusted with H_2_O to 50 µL. The blank contained no Phe (H_2_O instead). The samples were incubated at 37 °C for 1 h. The reaction was stopped by the addition of 150 µL of 1 M NaOH. The absorbance was read within five minutes at 322 nm (due to phenylpyruvate enol ε_322nm_ = 16,000 M^−1^ cm^−1^). Spectrophotometric determinations were conducted with a SpectraMax 96-well plate spectrophotometer (Molecular Devices, Sunnyvale, CA, USA).

### 4.8. Statistics

The project involved four groups of animals, 3–6 rats in each. For each animal, up to 64 measurements were performed (16 AAs in four types of biological samples). Some AAs were not included in the statistical processing due to implicit trends depending on the doses of TAA used. The relationship between the AA concentrations and their quantitative features in different types of samples with different doses of TAA (0, 200, 400, and 600 mg/kg) was studied. There were 192 independent hypotheses formulated of no difference between the concentrations of AAs in the rat groups (16 AAs, three TAA groups, and four types of samples) and 192 independent hypotheses of no difference between the test groups (16 AAs, three pairs of groups, and four types of samples). Overall, 384 initial hypotheses were involved in the analysis. A multivariate analysis of variance (MANOVA) and Tukey’s test were used to test the hypotheses. To correct for multiple-hypothesis testing, the Benjamini–Hochberg procedure was used. After the checks, a two-factor analysis of variance was performed for pairs of dependent variables.

## 5. Conclusions

A complete restoration of the balance of AAs in the blood plasma and tissues of rats 6 days after TAA intoxication was not revealed. In all rat samples, an imbalance was noted in a number of individual AAs, AA groups, as well as metabolites and enzymes of the Gln–Glu cycle.

The nature of the distribution of AAs in the blood plasma and tissues of TAA-induced rats indicated the presence of various dose-dependent recovery mechanisms.

The nature of the restoration of the balance of AAs in the blood plasma and tissues of rats after exposure to TAA indicated the presence of a certain affinity in the pairs “liver-brain” and “kidney-blood plasma”. The revealed changes in the AA balance in groups of TAA-induced rats in the postrehabilitation period may be useful for monitoring rehabilitation measures after acute HE.

## Figures and Tables

**Table 1:** Levels of amino acid in plasma and tissue of control (n = 3) and TAA-induced rats (n = 13, TAA at doses of 200–600 mg/kg). Plasma AA levels are expressed in µM and tissue AA levels are expressed in nmol per mg protein. Data are presented as mean ± standard deviation. ND—no data. Significantly different values compared to controls marked as (*) for *p* ≤ 0.1 and (**) for *p* ≤ 0.05.

AA	Plasma		Liver		Kidney		Brain	
	Control	TAA 200–600	Control	TAA 200–600	Control	TAA 200–600	Control	TAA 200–600
Aspartate	37.5 ± 0.76	34.6 ± 11.1	4.19 ± 0.76	3.30 ± 1.39	5.74 ± 2.86	6.87 ± 3.08	9.68 ± 3.58	7.40 ± 1.95
Glutamate	136 ± 22.7	123 ± 33.9	8.39 ± 2.49	8.91 ± 1.74	41.0 ± 8.63	47.6 ± 19.2	40.7 ± 15.0	30.1 ± 7.94
Glutamine	689 ± 70.7	816 ± 87.8	14.8 ± 2.83	22.2 ± 4.53 **	9.94 ± 3.68	12.4 ± 4.90	17.8 ± 5.50	14.78 ± 4.31
Glycine	316 ± 50.0	327 ± 44.3	7.33 ± 1.77	6.43 ± 1.15	13.6 ± 6.57	26.9 ± 10.6	2.05 ± 0.71	1.91 ± 0.41
Citrulline	233 ± 7.62	241 ± 31.0 *	3.61 ± 0.72	3.21 ± 0.59	NA	NA	1.31 ± 0.45	1.11 ± 0.26
Arginine	272 ± 35.7	311 ± 39.6	0.62 ± 0.04	0.73 ± 0.26	2.41 ± 1.87	1.40 ± 0.51	0.38 ± 0.10	0.39 ± 0.11
Taurine	207 ± 28.2	183 ± 25.6	3.57 ± 1.57	17.0 ± 14.4	42.6 ± 16.1	49.9 ± 23.4	15.4 ± 4.53	12.2 ± 3.56
Alanine	539 ± 82.9	532 ± 68.6	9.93 ± 1.94	8.09 ± 1.78	3.55 ± 1.76	6.39 ± 2.69	2.38 ± 0.71	2.14 ± 0.49
Tryptophan	266 ± 34.6	287 ± 42.6	0.39 ± 0.02	0.37 ± 0.08	1.66 ± 0.75	1.82 ± 0.82	0.24 ± 0.09	0.22 ± 0.08
Methionine	51.1 ± 11.7	69.8 ± 7.53 **	0.16 ± 0.01	0.23 ± 0.07 *	1.04 ± 1.01	0.49 ± 0.19	0.10 ± 0.05	0.09 ± 0.04
Valine	135 ± 22.3	144 ± 13.2	0.69 ± 0.03	0.83 ± 0.20	0.78 ± 0.28	4.08 ± 2.34	0.17 ± 0.06	0.18 ± 0.05
Phenylalanine	54.8 ± 2.86	60.7 ± 4.67 **	0.36 ± 0.02	0.44 ± 0.09	1.11 ± 1.03	0.38 ± 0.15	0.12 ± 0.04	0.11 ± 0.03
Isoleucine	89.5 ± 12.1	95.3 ± 10.1	0.66 ± 0.03	0.75 ± 0.17	1.42 ± 1.06	1.06 ± 0.39	0.14 ± 0.04	0.14 ± 0.03
Leucine	140 ± 21.9	150 ± 12.8	1.03 ± 0.04	1.29 ± 0.31	4.02 ± 3.58	1.88 ± 0.76	0.16 ± 0.05	0.22 ± 0.07 *
Ornithine	59.8 ± 10.7	73.8 ± 9.80	0.70 ± 0.07	1.16 ± 0.31 **	0.83 ± 0.50	1.00 ± 0.53	0.11 ± 0.01	0.12 ± 0.03
Lysine	539 ± 52.7	613 ± 52.2 *	2.03 ± 0.21	2.30 ± 0.57	5.23 ± 3.44	5.22 ± 2.54	0.64 ± 0.24	0.58 ± 0.16

## Data Availability

Additional data may be available upon personal request.

## Supplementary Materials

The supporting information can be downloaded at: https://www.mdpi.com/article/10.3390/ijms24043647/s1.
