# Supplementary material for: Residual Amino Acid Imbalance in Rats during Recovery from Acute Thioacetamide-Induced Hepatic Encephalopathy Indicates Incomplete Healing
Source: Int J Mol Sci. 2023 Feb 11;24(4):3647. doi: 10.3390/ijms24043647 (PMC9967446; doi:10.3390/ijms24043647)
Supplement: Supplementary file 1 [file ijms-24-03647-s001.zip › ijms-2185510-supplementary.pdf]

Supplement 1

Table 1. Calculated correlations (p-values, function ‘cor_test’) with a significance level ≤5% between AA variations in samples of the same type. *p*-values were obtained for all the pairs of AA concentrations (AA1 and AA2) for the same tissues.

**Brain tissue**			**Kidney tissue**			**Liver tissue**			**Blood plasma**		
AA1	AA2	p-value_corr	AA1	AA2	p-value_corr	AA1	AA2	p-value_corr	AA1	AA2	p-value_corr
asp_b	gly_b	0,001	asp_k	ala_k	0,001	met_l	phe_l	0,001	glu_p	cit_p	0,004
asp_b	met_b	0,001	asp_k	trp_k	0,001	iso_l	orn_l	0,001	iso_p	leu_p	0,004
asp_b	val_b	0,001	gln_k	gly_k	0,001	ala_l	trp_l	0,002	asp_p	tau_p	0,007
glu_b	gly_b	0,001	gln_k	trp_k	0,001	orn_l	lys_l	0,002	val_p	leu_p	0,009
glu_b	tau_b	0,001	gln_k	val_k	0,001	arg_l	orn_l	0,003	glu_p	tau_p	0,011
gln_b	gly_b	0,001	gly_k	tau_k	0,001	trp_l	iso_l	0,003	cit_p	phe_p	0,015
gly_b	trp_b	0,001	trp_k	iso_k	0,001	trp_l	val_l	0,004	gly_p	cit_p	0,022
gly_b	met_b	0,001	asp_k	gly_k	0,002	gln_l	leu_l	0,005	asp_p	cit_p	0,034
gly_b	lys_b	0,001	asp_k	val_k	0,002	phe_l	lys_l	0,005	glu_p	gly_p	0,05
cit_b	trp_b	0,001	tau_k	val_k	0,002	gln_l	met_l	0,006	phe_p	orn_p	0,05
arg_b	met_b	0,001	iso_k	lys_k	0,002	phe_l	orn_l	0,006	n=10, average *p*-value=0.021		
tau_b	val_b	0,001	glu_k	val_k	0,003	gln_l	val_l	0,008			
trp_b	iso_b	0,001	iso_k	orn_k	0,006	arg_l	met_l	0,01			
val_b	leu_b	0,001	glu_k	iso_k	0,008	gln_l	iso_l	0,011			
asp_b	iso_b	0,002	gly_k	trp_k	0,008	gln_l	orn_l	0,011			
glu_b	val_b	0,002	ala_k	trp_k	0,008	gln_l	phe_l	0,013			
asp_b	trp_b	0,004	asp_k	iso_k	0,01	trp_l	leu_l	0,013			
glu_b	lys_b	0,004	ala_k	lys_k	0,012	trp_l	lys_l	0,013			
glu_b	iso_b	0,005	met_k	lys_k	0,012	arg_l	lys_l	0,019			
glu_b	met_b	0,006	val_k	lys_k	0,012	trp_l	met_l	0,02			
gln_b	arg_b	0,009	glu_k	orn_k	0,014	arg_l	val_l	0,028			
arg_b	tau_b	0,009	gly_k	lys_k	0,02	trp_l	phe_l	0,032			
arg_b	phe_b	0,009	tau_k	iso_k	0,02	arg_l	leu_l	0,037			
phe_b	leu_b	0,009	gln_k	iso_k	0,022	gln_l	lys_l	0,038			
met_b	leu_b	0,015	tau_k	orn_k	0,024	cit_l	trp_l	0,048			
cit_b	leu_b	0,022	arg_k	orn_k	0,025	gly_l	trp_l	0,05			
gly_b	leu_b	0,023	trp_k	val_k	0,025	n=26, average *p*-value=0.015					
glu_b	trp_b	0,024	trp_k	met_k	0,028						
gly_b	arg_b	0,025	arg_k	trp_k	0,029						
trp_b	leu_b	0,029	orn_k	lys_k	0,038						
leu_b	lys_b	0,036	arg_k	lys_k	0,042						
arg_b	val_b	0,037	leu_k	lys_k	0,044						
arg_b	iso_b	0,048	n=32, average *p*-value=0.013								
asp_b	arg_b	0,05									
n=34, average *p*-value=0.011											

Table 2. ANOVA *p*-values and Tukey test adjusted *p*-values for 10 AA.

AA	Tissue	ANOVA *p*-value	Groups of comparison and corresponding adjusted *p*-values					
			0 vs 200	0 vs 400	0 vs 600	200 vs 400	200 vs 600	400 vs 600
Methionine	Liver	0,022	0,990	0,077	0,214	0,042	0,130	0,953
Lysine	Liver	0,023	0,596	0,580	0,207	0,070	0,021	0,717
Ornithine	Liver	0,029	0,862	0,089	0,039	0,350	0,157	0,852
Glutamine	Liver	0,048	0,465	0,032	0,312	0,469	0,997	0,528
Leucine	Liver	0,049	0,980	0,251	0,191	0,127	0,099	0,979
Phenylalanine	Plasma	0,024	0,017	0,283	0,641	0,168	0,089	0,912
Citrulline	Plasma	0,029	0,089	0,999	0,974	0,039	0,032	0,973
Methionine	Plasma	0,037	0,042	0,059	0,079	0,909	0,937	1,000
Lysine	Plasma	0,083	0,057	0,417	0,351	0,342	0,542	0,990
Leucine	Brain	0,048	0,952	0,907	0,060	0,999	0,153	0,086

(yellow) *p*-value less then 5%
(green) *p*-value less then 10%

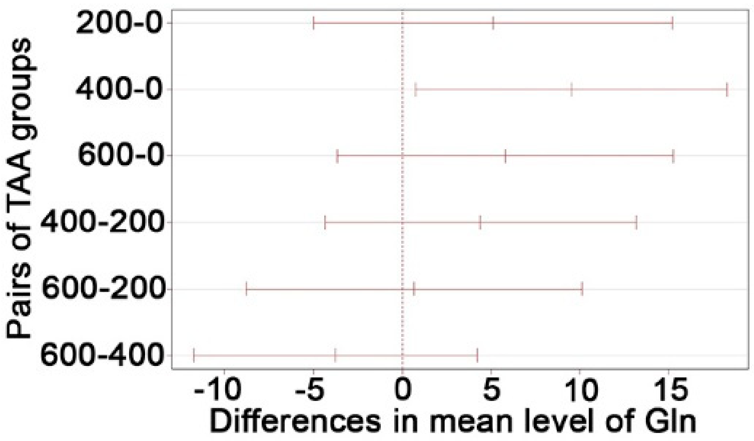

Figure 1. Tukey simultaneous 95% confidence intervals for Gln in liver.

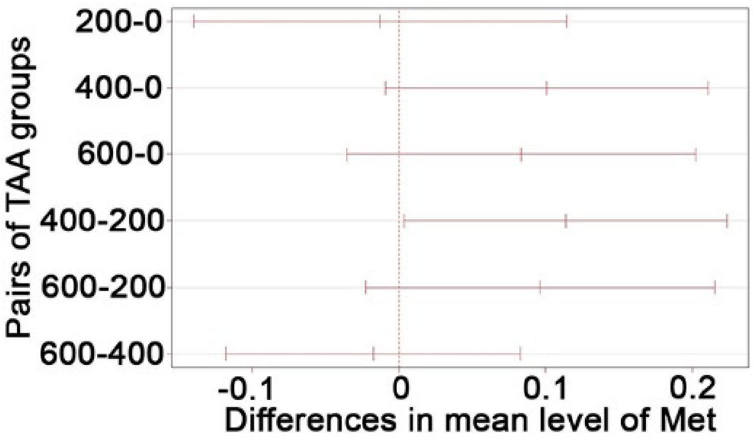

Figure 2. Tukey simultaneous 95% confidence intervals for Met in liver.

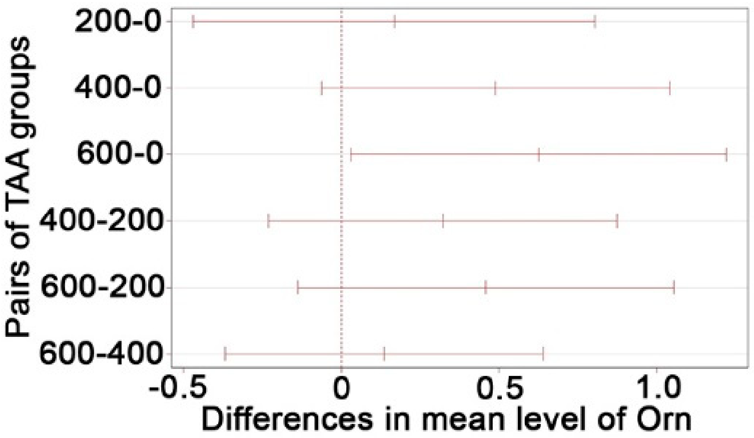

Figure 3. Tukey simultaneous 95% confidence intervals for Orn in liver.

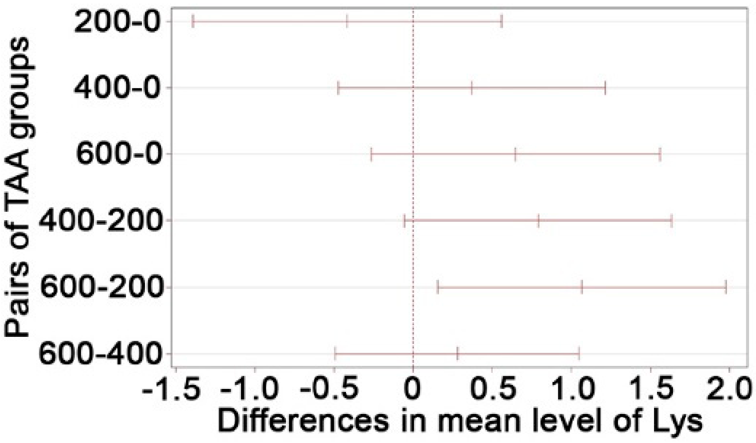

Figure 4. Tukey simultaneous 95% confidence intervals for Lys in liver.

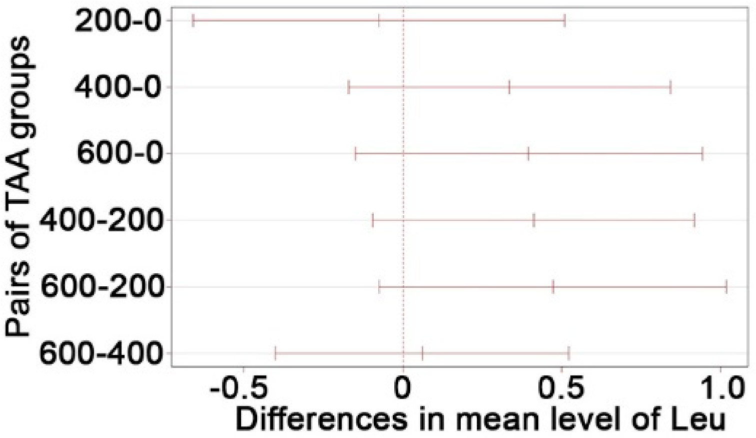

Figure 5. Tukey simultaneous 95% confidence intervals for Leu in liver.

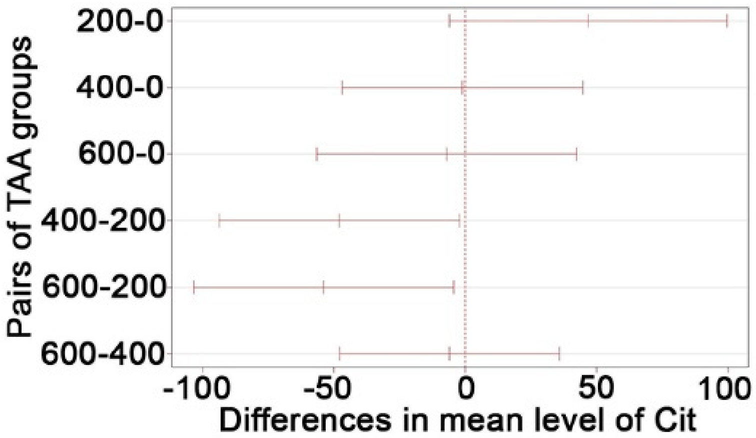

Figure 6. Tukey simultaneous 95% confidence intervals for Cit in plasma.

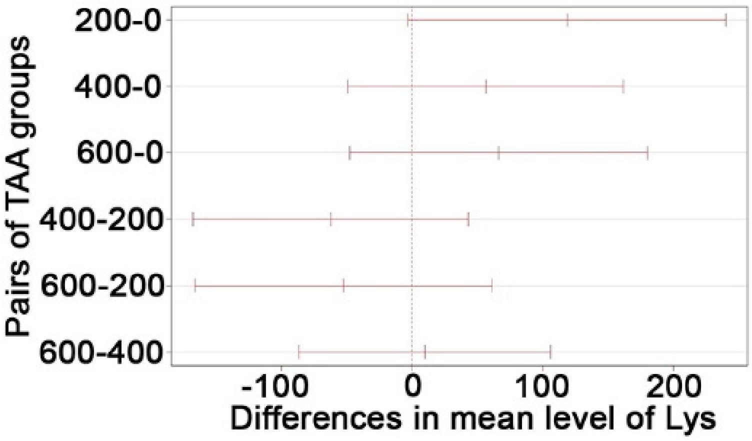

Figure 7. Tukey simultaneous 95% confidence intervals for Lys in plasma.

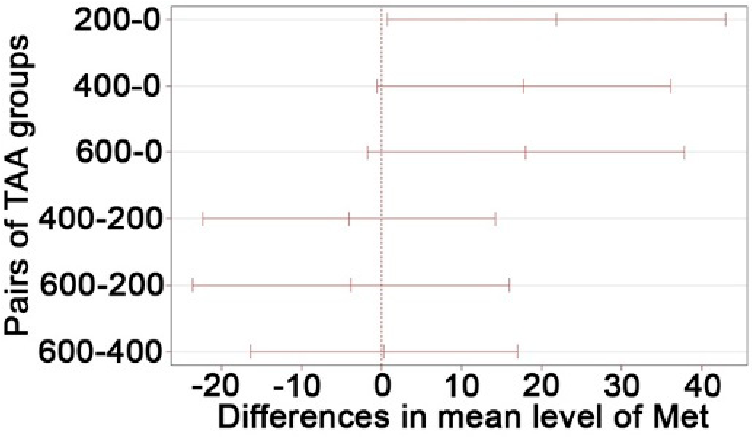

Figure 8. Tukey simultaneous 95% confidence intervals for Met in plasma.

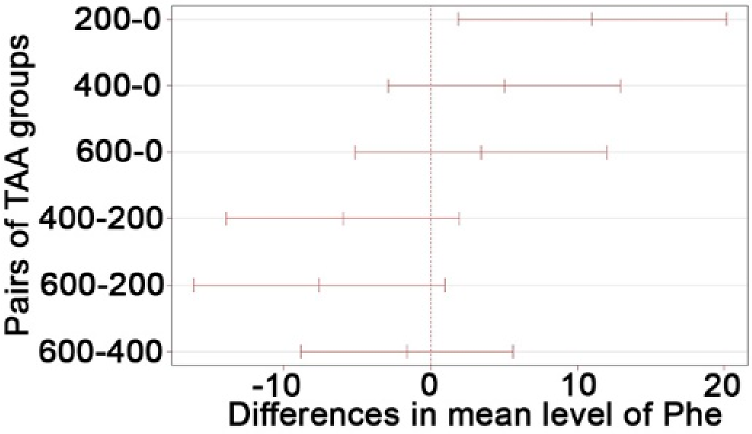

Figure 9. Tukey simultaneous 95% confidence intervals for Phe in plasma.

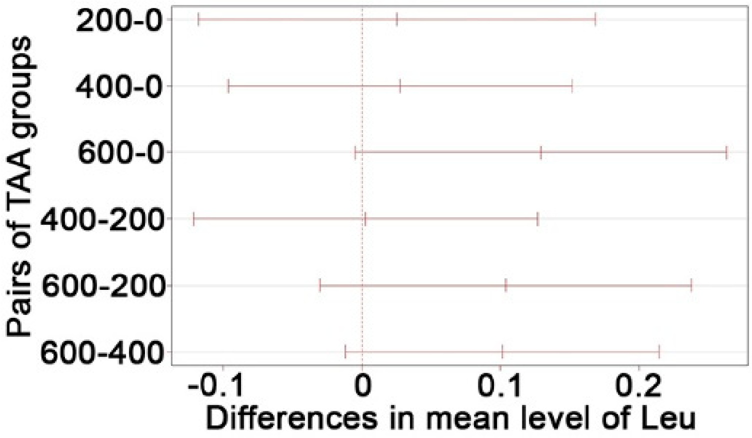

Figure 10. Tukey simultaneous 95% confidence intervals for Leu in brain.
